# Feasibility Evaluation of Commercially Available Video Conferencing Devices to Technically Direct Untrained Nonmedical Personnel to Perform a Rapid Trauma Ultrasound Examination

**DOI:** 10.3390/diagnostics9040188

**Published:** 2019-11-14

**Authors:** Davinder Ramsingh, Michael Ma, Danny Quy Le, Warren Davis, Mark Ringer, Briahnna Austin, Cameron Ricks

**Affiliations:** 1 Department of Anesthesiology, Loma Linda University Health, 11234 Anderson St. MC-2532, Loma Linda, CA 92354, USA; 2 Department of Anesthesiology, UCI Medical Center, Orange, CA 92868, USA; ma.michael@gmail.com (M.M.); cricks@uci.edu (C.R.); 3 David Geffen School of Medicine at UCLA, Los Angeles, CA 90095, USA; dannyle097@gmail.com; 4 Department of Anesthesiology, St. Joseph Medical Center, 7601 Osler Drive, Towson, MD 21204, USA; wardav@gmail.com; 5 Loma Linda University School of Medicine, Loma Linda, CA 92350, USA; mringer@llu.edu

**Keywords:** point-of-care ultrasound, telemedicine, medical education

## Abstract

**Introduction:** Point-of-care ultrasound (POCUS) is a rapidly expanding discipline that has proven to be a valuable modality in the hospital setting. Recent evidence has demonstrated the utility of commercially available video conferencing technologies, namely, FaceTime (Apple Inc, Cupertino, CA, USA) and Google Glass (Google Inc, Mountain View, CA, USA), to allow an expert POCUS examiner to remotely guide a novice medical professional. However, few studies have evaluated the ability to use these teleultrasound technologies to guide a nonmedical novice to perform an acute care POCUS examination for cardiac, pulmonary, and abdominal assessments. Additionally, few studies have shown the ability of a POCUS-trained cardiac anesthesiologist to perform the role of an expert instructor. This study sought to evaluate the ability of a POCUS-trained anesthesiologist to remotely guide a nonmedically trained participant to perform an acute care POCUS examination. **Methods:** A total of 21 nonmedically trained undergraduate students who had no prior ultrasound experience were recruited to perform a three-part ultrasound examination on a standardized patient with the guidance of a remote expert who was a POCUS-trained cardiac anesthesiologist. The examination included the following acute care POCUS topics: (1) cardiac function via parasternal long/short axis views, (2) pneumothorax assessment via pleural sliding exam via anterior lung views, and (3) abdominal free fluid exam via right upper quadrant abdominal view. Each examiner was given a handout with static images of probe placement and actual ultrasound images for the three views. After a brief 8 min tutorial on the teleultrasound technologies, a connection was established with the expert, and they were guided through the acute care POCUS exam. Each view was deemed to be complete when the expert sonographer was satisfied with the obtained image or if the expert sonographer determined that the image could not be obtained after 5 min. Image quality was scored on a previously validated 0 to 4 grading scale. The entire session was recorded, and the image quality was scored during the exam by the remote expert instructor as well as by a separate POCUS-trained, blinded expert anesthesiologist. **Results:** A total of 21 subjects completed the study. The average total time for the exam was 8.5 min (standard deviation = 4.6). A comparison between the live expert examiner and the blinded postexam reviewer showed a 100% agreement between image interpretations. A review of the exams rated as three or higher demonstrated that 87% of abdominal, 90% of cardiac, and 95% of pulmonary exams achieved this level of image quality. A satisfaction survey of the novice users demonstrated higher ease of following commands for the cardiac and pulmonary exams compared to the abdominal exam. **Conclusions:** The results from this pilot study demonstrate that nonmedically trained individuals can be guided to complete a relevant ultrasound examination within a short period. Further evaluation of using telemedicine technologies to promote POCUS should be evaluated.

## 1. Introduction

Historically, the development of technology in medicine has more often been at the burden of higher costs. Indeed, recent reports suggest that, despite its increasing use, the cost of medical technology is not decreasing [1]. However, two areas in medicine in which this has been disproven are the categories of telemedicine and portable ultrasound.

Early ultrasound devices were large and often confined to hospital facilities that support imaging laboratories (cardiology, radiology, and obstetrics). With recent advances in ultrasound technology, however, these devices have become more portable, smaller, cheaper, and usable at the patient’s bedside [2]. Indeed, point-of-care ultrasound (POCUS) has been identified as the most rapidly growing sector in medical ultrasound imaging, with handheld devices costing approximately 1/20th the price of 10 years ago (from $40,000 + to $2000) [3]. In addition, ultrasound provides the particular benefit of a significantly lower level of harm compared to other medical imaging modalities [4].

Point-of-care ultrasound refers to the use of portable ultrasonography at the patient’s bedside for diagnostic and therapeutic purposes [5]. POCUS has been proven to serve a vital role in the rapid assessment of a patient’s cardiac, pulmonary, hemodynamic, vascular, neurologic, and gastrointestinal status [4,6,7,8]. Additionally, the application of POCUS continues to broaden as its usefulness and efficiency are shown to be greater than alternative imaging [4,9]. While the clinical utility of POCUS is rapidly expanding, the majority of the evidence supports its utility by skilled practitioners with advanced medical training. However, with innovations in POCUS devices described above, the possibility of using this technology in settings where skilled practitioners are not available becomes more feasible. Indeed, the barrier to using POCUS in resource-limited settings may be secondary to a lack of education rather than the availability of equipment.

The area of telemedicine has also undergone a similar transformation to POCUS over recent years. Telemedicine is defined as the use of medical information exchanged from one site to another via electronic communications to improve patients’ health status. The incorporation of consumer-level products has significantly reduced costs and has allowed this topic to become much more mainstream [10]. Indeed, consumer devices are demonstrating adequate capability for performing remote patient examinations, with continued improvements each year [10]. Furthermore, these technologies have advanced to allow hands-free video communication, with the remote viewer having a point-of-view (POV) perspective [11]. This innovation opens up new possibilities for application toward patient care.

Recently, consumer-available wearable technologies, such as Google Glass (GG; Mountain View, CA, USA), and routine video conferencing smartphone devices, such as Apple iPhone (Cupertino, CA, USA), have demonstrated utility in healthcare. The GG POV technology has demonstrated utility for improving intraoperative communication and documentation as well as surgical training [12]. The use of the FaceTime (FT) video conferencing technology from iPhone has demonstrated utility across a wide area of telemedicine applications. An abbreviated review demonstrated FT-supported telemedicine to be useful for primary care patient evaluation [13], dermatology evaluation [14], and management in the intensive care unit [15] as well as for improving the education of ultrasound-guided anesthetic procedures [16] and even airway management [17].

The integration of these two rapidly advancing areas (POCUS and telemedicine) has been explored. Recent evidence has demonstrated the ability of a POCUS expert physician to guide a nonphysician hospital staff member to perform a POCUS exam via consumer-available teleconference equipment [9]. This same research group also demonstrated the ability of FT technology to transfer ultrasound images without clinically significant quality degradation [18]. Moreover, the use of FT communication between an intensivist team at a tertiary care center and nonphysician healthcare providers in a low-income country demonstrated the ability to successfully educate about POCUS image acquisition techniques as well allow appropriate image quality for remote clinical interpretation [10]. Additionally, Zennaro et al. demonstrated a high degree of agreement between ultrasound exams performed by pediatricians who were guided remotely by radiologists and ultrasound exams performed by radiologists in person [19]. Similar results were found for the use of ultrasound with telemedicine (teleultrasound) for remote guidance between onsite resident physicians and remote expert mentors for the diagnosis of pediatric acute appendicitis [20]. Recently, the incorporation of augmented reality has also demonstrated utility for remote ultrasound guidance [21]. Finally, the development of a teleultrasound system that allows an expert to perform an ultrasound exam remotely via a robotic system has also demonstrated clinical utility [22].

While the utilization of teleultrasound has rapidly increased across many specialties for in-hospital patient care, far less has been explored for the use of POCUS technology to facilitate the management of patients in the out-of-hospital setting. Moreover, while the efficacy of teleultrasound between medical personnel has demonstrated patient benefit, there is much less evidence on the utility of implementing teleultrasound between a POCUS-trained physician and a person without medical training. Given the advancements in teleultrasound technology, there may be a potential to use these devices in resource-limited areas in which significant medical training is not available.

This scenario demonstrates the utility of evaluating the use of a low-cost teleultrasound system to guide nonmedical personnel to perform POCUS exams remotely. This pilot study sought to evaluate the ability of a POCUS-trained physician to remotely guide nonmedical personnel to perform an acute cardiac, pulmonary, and abdominal POCUS exam using consumer-available communication devices, namely, FT and GG. Specifically, this blinded study sought to evaluate the quality of images captured by untrained, nonmedical, tele-ultrasound-guided sonographers in comparison to the quality of images performed by an expert sonographer.

## 2. Methods

### 2.1. Study Design

This prospective, educational intervention study was approved by the Institutional Review Board (IRB # # 2014-1014) at the University of California—Irvine, Orange CA on 21 February 2014.

### 2.2. Population and Setting

Subjects were undergraduate students at the University of California, Irvine, who voluntarily consented to participate and who confirmed to having no medical ultrasound training.

### 2.3. Point-of-Care Ultrasound Exam

**Figure 1:**
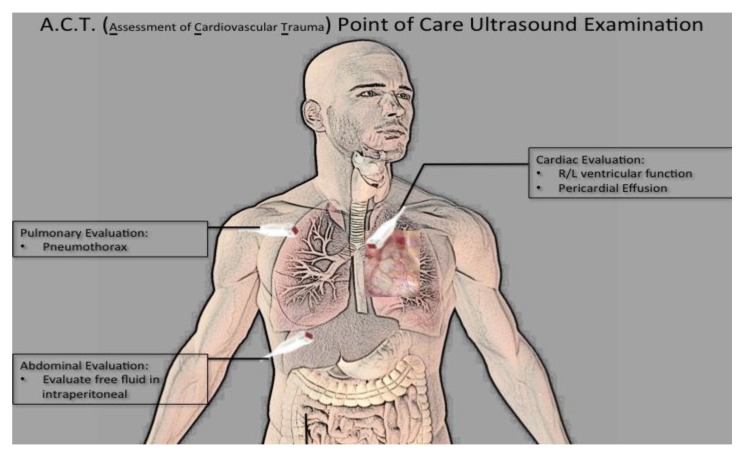
Focused ultrasound examination for assessment of cardiovascular trauma.

A previously validated point-of-care ultrasound was designed for the assessment of cardiovascular trauma (ACT) and included (1) cardiac evaluation via parasternal long and short-axis views, (2) pulmonary evaluation for pneumothorax via anterior lung sliding evaluation, and (3) abdominal free fluid evaluation via right upper quadrant scan (Figure 1) [23]. A high-frequency transducer (12 mHz) was used for pulmonary evaluation, and a low-frequency (2–5 mHz) transducer was used for cardiac and abdominal evaluation.

### 2.4. Experimental Protocol

After consent, participants received an 8 min tutorial on the following: ultrasound equipment (how to switch ultrasound probes, adjust depth, adjust gain, and locate the probe indicator), Google Glass (how to wear and connect audio), and iPhone FT video conferencing technology. The subjects also received a one-page handout that showed an image of correct probe placement on the body as well as an ideal ultrasound image for each of the three components of the ACT exam. No additional instruction on the ultrasound exam, including probe orientation, anatomy, image quality, or image interpretation, was performed as the study examined the ability to guide a true novice. After the tutorial, the subject ultrasonographer donned the GG, and a connection was established with the expert sonographer stationed in another room. FaceTime connection was also established; however, this device was positioned to show only the ultrasound screen at all times. The expert anesthesiologist visualized both the FT and GG streams on a notebook computer (MacBook Pro 2013). Goggle Glass provided a one-way video and two-way audio communication. FaceTime provided two-way audio and video communication. The expert instructed the ultrasonographer via the GG device.

A SonoSite (Bothell, WA) Edge I system with a 12 MHz linear transducer and a 2–5 MHz phased-array transducer was used for all ultrasound exams. All exams were performed on the same human model, who was a young, healthy male with normal body habitus. A research assistant was present to support any technical issues with communication devices or the ultrasound equipment. Each subject had up to 5 min to complete each of the three ultrasound exams. A five-point scale, as described previously [24], was used, with the value of 3 representing the cut-off score for images deemed suitable for interpretation. The remote expert examiner would stop the subject after an ideal image was achieved (based on the remote examiner’s judgment for having an image score ≥3) or if the 5 min exam period elapsed. The expert examiner would score the live image quality for each of three exams immediately after image acquisition.

Additionally, the audio and video feed on the expert examiner’s computer was recorded for offline review by a blinded second expert examiner. Prior to study initiation, the blinded second examiner and the expert study sonographer reviewed five complete ACT exam clips to validate inter-rater scoring reliability. Additionally, the blinded reviewer reviewed the clips of the expert sonographer performing an in-person exam on the model used for this study, taking them as the benchmark for the highest-quality image (5/5). No participants were compensated in any way.

### 2.5. Primary Outcome Measure

For each of the three components of the ACT exam, the frequency of obtaining an adequate image quality, defined as having a value of 3 or higher, was measured. The goal was to identify adequate image quality for at least 80% of the exams.

### 2.6. Secondary Outcome Measures

All components of the exams were recorded and compared to the exam time of the expert sonographer’s in-person examination. All subjects completed surveys on the ease of use of the telecommunication system and the teleultrasound process. The model also completed surveys regarding the level of comfort during the examination for all examiners. The experience surveys of the model were compared between the subjects and the expert sonographer.

### 2.7. Sample Size and Statistics

Estimation of sample size was based on a priori assumption of a 40% image acquisition rate of clinically interpretable images (>3/5) of the nonmedical participants without teleultrasound assistance. A sample size of 20 subjects was calculated to increase the image acquisition rate to the target of 80%, assuming a power of 0.80 and alpha 0.05. Descriptive statistics were used to report the image acquisition rate for each of the three components of the ACT exam.

## 3. Results

**Figure 2:**
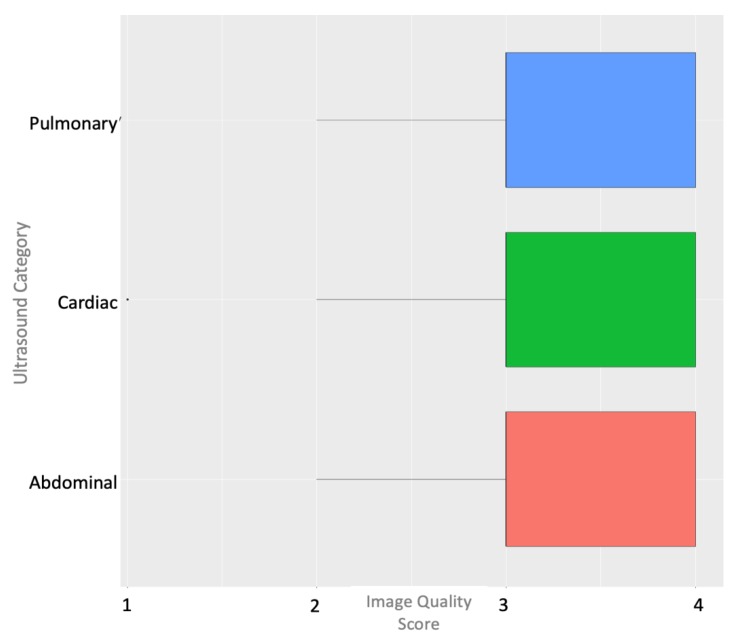
Box plot of image quality scores by ultrasound category.

**Figure 3:**
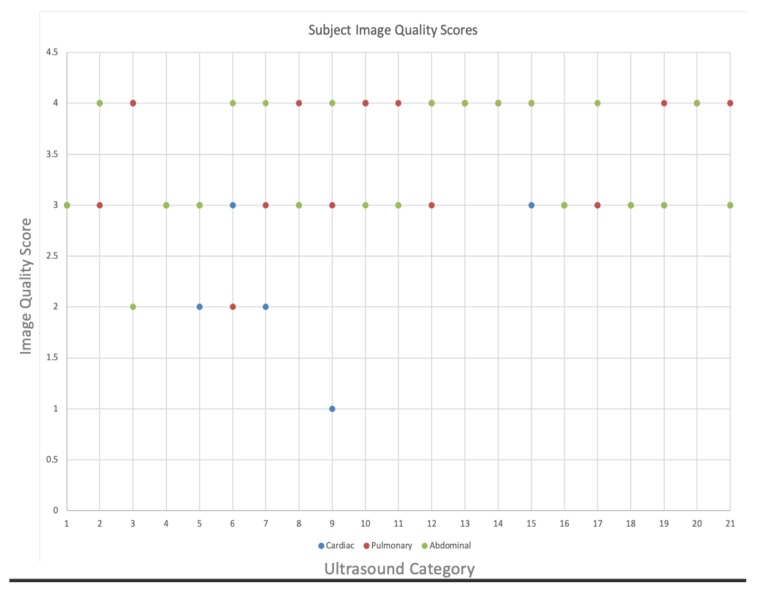
Complete subject image quality scores.

A total of 21 nonmedically trained undergraduate students without prior ultrasound experience were recruited and completed the study. The average time to complete the specific exams was as follows: cardiac exam, mean = 4.7 (standard deviation (SD) = 3.6 min); pulmonary exam, mean = 1.6 (SD = 1.2 min); abdominal exam, mean = 2.1 (SD = 1.6 min). The average total time for the exam was 8.5 min (SD = 4.6). A comparison between the live expert examiner and the blinded postexam reviewer showed a 100% agreement between image interpretations. A review of the exams rated as three or greater demonstrated that 87% of abdominal, 90% of cardiac, and 95% of pulmonary exams achieved this level of image quality (Figure 2). The complete distribution of the image quality results for each exam type is shown in Figure 3. Standardized patient comfort survey results showed 5/5 scores for all ultrasound examinations. Summary data from the satisfaction survey of the teleultrasound communication by the novice users was as follows on a 5-point Likert scale: audio quality, median = 3.0 (interquartile range (IQR) = 2); ease of following commands, median = 5 (IQR = 1); comfort in obtaining cardiac view, median = 5 (IQR = 1); comfort in obtaining lung view, median = 5 (IQR = 1); and comfort in obtaining abdominal view, median = 4 (IQR = 1).

## 4. Discussion

This pilot study suggests that nonmedical individuals can be directed to complete an acute care POCUS exam using the combination of a POV hands-free audio-visual device (GG) with a commercially available smartphone video conferencing platform (FT). As the technology and cost of medical ultrasound continue to improve, the expansion of its role in healthcare needs to be explored. By combining POCUS technologies with commercially available video communication technologies, one can evaluate new areas to implement portable ultrasound.

Multiple studies have demonstrated the ability to use teleultrasound to connect healthcare providers across vast distances and between rural areas and those in larger medical centers [25]. For example, the ability to instruct paramedics to perform a trauma POCUS examination via real-time physician guidance has been demonstrated [26]. Additionally, Sheehan et al. demonstrated the ability to remotely guide inexperienced users through an ultrasound examination with the application of a visual guidance system [27]. Indeed, the ability to perform POCUS exams in space via remote guidance has also been proven [28]. Further efforts have continued to demonstrate that teleultrasound allows end-users to perform ultrasound examinations with minimal training [25] and that these examinations generate clinically useful ultrasound images for a variety of organ systems and pathologies [25].

As teleultrasound continues to grow, exploration of how this concept can be applied using low-cost, commercially available products have recently been explored. Multiple studies have demonstrated the utility of smartphone-based video conferencing platforms to remotely instruct POCUS examinations [10,16,29]. Images obtained from these platforms are noninferior to those obtained directly from the ultrasound device [18]. In addition, wearable video conferencing devices, such as GG, have also been applied to POCUS applications. For example, the utility of GG for assistance with central venous access has been demonstrated [30].

However, few studies have examined the combination of a wearable video conferencing device (GG) and a smartphone video conferencing platform (FT) to remotely guide nonmedical participants to perform an acute care POCUS examination. This combination is novel and provides a hands-free strategy to provide remote guidance. Additionally, this study demonstrates a cost-effective teleultrasound system that can provide successful remote guidance as well as real-time remote image interpretation. Moreover, our data supports that the time to perform these examinations is practical for acute care management. Indeed, with the rapid cost reduction in teleultrasound technologies and the data demonstrated in our study, further research should explore ways to apply POCUS in new patient care settings. For instance, many public health and international medical mission trips include personnel without medical training. Teleultrasound may increase the contribution of these individuals.

Additionally, this study is also one of the first demonstrating the ability of a perioperative physician to facilitate the use of ultrasound technology outside of the hospital setting. Within the limits of this small, simulation-based study, our results suggest that an appropriately trained anesthesiologist can effectively provide guidance via the described teleultrasound system. This is relevant given the continuous pressure on the specialty of anesthesiology to expand its role in the patient care continuum.

While this study did not assess the application of this system toward patient care, our positive results support the need for additional studies in this area. Specifically, how these low-cost technologies can be used to improve the quality of care in resource-limited environments should be evaluated.

This study has several limitations. It is a small pilot project designed to evaluate the feasibility of a POCUS-trained anesthesiologist to use commercially available teleultrasound technologies. Future studies should evaluate this utility over a larger sample size that is applied over a region more common to traditional telemedicine platforms. In addition, all studies were performed on the same model (both by the expert and participants) to remove the confounding variable of different acoustic windows in patients. No evaluation of pathology was assessed in the study; rather, the focus was on the ability to generate an image that was interpretable remotely with the described teleultrasound technology. Finally, the participants were not tested on the anatomy or interpretation of the image.

## 5. Conclusions

This pilot project demonstrated the utility of a novel teleultrasound system to guide nonmedically trained adults to successfully acquire ultrasound images useful for acute cardiac, pulmonary, and abdominal assessments. This novel system utilizes low-cost, commercially available technologies and allows the examiner to communicate hands-free. Further studies should evaluate how this described system can be utilized to expand the role of teleultrasound.

## References

[1] Kumar R.K. (2011). Technology and healthcare costs. Ann. Pediatr. Cardiol..

[2] Alpert J.S., Mladenovic J., Hellmann D.B. (2009). Should a hand-carried ultrasound machine become standard equipment for every internist?. Am. J. Med..

[3] Prescient & Strategic Intelligence Point-of-Care Ultrasound (Pocus) Device Market to Grow at 6.9% Cagr Till 2025: P&s Market Research. Https://globenewswire.Com/news-release/2017/07/21/1055557/0/en/point-of-care-ultrasound-pocus-device-market-to-grow-at-6-9-cagr-till-2025-p-s-market-research.Html.

[4] Moore C., Copel J. (2011). Point-of-care ultrasonography. N. Engl. J. Med..

[5] Kendall J.L., Hoffenberg S.R., Smith R.S. (2007). History of emergency and critical care ultrasound: The evolution of a new imaging paradigm. Crit. Care Med..

[6] Arntfield R.T., Millington S.J. (2012). Point of care cardiac ultrasound applications in the emergency department and intensive care unit—A review. Curr. Cardiol. Rev..

[7] Manno E., Navarra M., Faccio L., Motevallian M., Bertolaccini L., Mfochive A., Pesce M., Evangelista A. (2012). Deep impact of ultrasound in the intensive care unit: The “icu-sound” protocol. Anesthesiology.

[8] Ramsingh D., Rinehart J., Kain Z., Strom S., Canales C., Alexander B., Capatina A., Ma M., Le K.V., Cannesson M. (2015). Impact assessment of perioperative point-of-care ultrasound training on anesthesiology residents. Anesthesiology.

[9] Levine A.R., McCurdy M.T., Zubrow M.T., Papali A., Mallemat H.A., Verceles A.C. (2015). Tele-intensivists can instruct non-physicians to acquire high-quality ultrasound images. J. Crit. Care.

[10] Robertson T.E., Levine A.R., Verceles A.C., Buchner J.A., Lantry J.H., Papali A., Zubrow M.T., Colas L.N., Augustin M.E., McCurdy M.T. (2017). Remote tele-mentored ultrasound for non-physician learners using facetime: A feasibility study in a low-income country. J. Crit. Care.

[11] Armstrong D.G., Giovinco N., Mills J.L., Rogers L.C. (2011). Facetime for physicians: Using real time mobile phone-based videoconferencing to augment diagnosis and care in telemedicine. Eplasty.

[12] Chang J.Y., Tsui L.Y., Yeung K.S., Yip S.W., Leung G.K. (2016). Surgical vision: Google glass and surgery. Surg. Innov..

[13] Robinson M.D., Branham A.R., Locklear A., Robertson S., Gridley T. (2016). Measuring satisfaction and usability of facetime for virtual visits in patients with uncontrolled diabetes. Telemed. J. E-Health.

[14] Wu X., Oliveria S.A., Yagerman S., Chen L., DeFazio J., Braun R., Marghoob A.A. (2015). Feasibility and efficacy of patient-initiated mobile teledermoscopy for short-term monitoring of clinically atypical nevi. JAMA Derm..

[15] Williams G.W., Buendia F.I., Idowu O.O. (2015). Utilization of a mobile videoconferencing tool (facetime) for real-time evaluation of critically ill neurosurgical patients. J. Neurosurg. Anesthesiol..

[16] Miyashita T., Iketani Y., Nagamine Y., Goto T. (2014). Facetime((r)) for teaching ultrasound-guided anesthetic procedures in remote place. J. Clin. Monit. Comput..

[17] Van Oeveren L., Donner J., Fantegrossi A., Mohr N.M., Brown C.A. (2017). Telemedicine-assisted intubation in rural emergency departments: A national emergency airway registry study. Telemed. J. E-Health.

[18] Levine A.R., Buchner J.A., Verceles A.C., Zubrow M.T., Mallemat H.A., Papali A., McCurdy M.T. (2016). Ultrasound images transmitted via facetime are non-inferior to images on the ultrasound machine. J. Crit. Care.

[19] Zennaro F., Neri E., Nappi F., Grosso D., Triunfo R., Cabras F., Frexia F., Norbedo S., Guastalla P., Gregori M. (2016). Real-time tele-mentored low cost “point-of-care us” in the hands of paediatricians in the emergency department: Diagnostic accuracy compared to expert radiologists. PLoS ONE.

[20] Kim C., Kang B.S., Choi H.J., Lim T.H., Oh J., Chee Y. (2015). Clinical application of real-time tele-ultrasonography in diagnosing pediatric acute appendicitis in the ed. Am. J. Emerg. Med..

[21] Anton D., Kurillo G., Yang A.Y., Bajcsy R. Augmented Telemedicine Platform for Real-Time Remote Medical Consultation. Proceedings of the 23rd International Conference (MMM).

[22] Avgousti S., Panayides A.S., Christoforou E.G., Argyrou A., Jossif A., Masouras P., Vieyres P. Medical telerobotics and the remote ultrasonography paradigm over 4g wireless networks. Proceedings of the 2018 IEEE 20th International Conference on e-Health Networking, Applications and Services (Healthcom).

[23] Ramsingh D., Alexander B., Le K., Williams W., Canales C., Cannesson M. (2014). Comparison of the didactic lecture with the simulation/model approach for the teaching of a novel perioperative ultrasound curriculum to anesthesiology residents. J. Clin. Anesth..

[24] Jakobsen C.J., Torp P., Sloth E. (2007). Perioperative feasibility of imaging the heart and pleura in patients with aortic stenosis undergoing aortic valve replacement. Eur. J. Anaesthesiol..

[25] Britton N., Miller M.A., Safadi S., Siegel A., Levine A.R., McCurdy M.T. (2019). Tele-ultrasound in resource-limited settings: A systematic review. Front. Public Health.

[26] Boniface K.S., Shokoohi H., Smith E.R., Scantlebury K. (2011). Tele-ultrasound and paramedics: Real-time remote physician guidance of the focused assessment with sonography for trauma examination. Am. J. Emerg. Med..

[27] Sheehan F.H., Ricci M.A., Murtagh C., Clark H., Bolson E.L. (2010). Expert visual guidance of ultrasound for telemedicine. J. Telemed. Telecare.

[28] Sargsyan A.E., Hamilton D.R., Jones J.A., Melton S., Whitson P.A., Kirkpatrick A.W., Martin D., Dulchavsky S.A. (2005). Fast at mach 20: Clinical ultrasound aboard the international space station. J. Trauma Acute Care Surg..

[29] Smith A., Addison R., Rogers P., Stone-McLean J., Boyd S., Hoover K., Pollard M., Dubrowski A., Parsons M. (2018). Remote mentoring of point-of-care ultrasound skills to inexperienced operators using multiple telemedicine platforms: Is a cell phone good enough?. J. Ultrasound Med..

[30] Wu T.S., Dameff C.J., Tully J.L. (2014). Ultrasound-guided central venous access using google glass. J. Emerg. Med..

